# Analysis of Magnetic Field Measurements for Indoor Positioning †

**DOI:** 10.3390/s22114014

**Published:** 2022-05-25

**Authors:** Guanglie Ouyang, Karim Abed-Meraim

**Affiliations:** Laboratoire Pluridisciplinaire de Recherche en Ingénierie des Systèmes, Mécanique et Energétique, Université d’Orléans, 12 Rue de Blois, 45067 Orleans, France; karim.abed-meraim@univ-orleans.fr

**Keywords:** magnetic field, smartphone, magnetometer calibration, indoor positioning

## Abstract

Infrastructure-free magnetic fields are ubiquitous and have attracted tremendous interest in magnetic field-based indoor positioning. However, magnetic field-based indoor positioning applications face challenges such as low discernibility, heterogeneous devices, and interference from ferromagnetic materials. This paper first analyzes the statistical characteristics of magnetic field (MF) measurements from heterogeneous smartphones. It demonstrates that, in the absence of disturbances, the MF measurements in indoor environments follow a Gaussian distribution with temporal stability and spatial discernibility. It shows the fluctuations in magnetic field intensity caused by the rotation of a smartphone around the Z-axis. Secondly, it suggests that the RLOWESS method can be used to eliminate magnetic field anomalies, using magnetometer calibration to ensure consistent MF measurements in heterogeneous smartphones. Thirdly, it tests the magnetic field positioning performance of homogeneous and heterogeneous devices using different machine learning methods. Finally, it summarizes the feasibility/limitations of using only MF measurement for indoor positioning.

## 1. Introduction

The global indoor positioning market size is expected to grow at a Compound Annual Growth Rate of 22.5%, from USD 6.1 billion in 2020 to USD 17 billion by 2025 [1]. Advanced indoor positioning technologies that can obtain accurate location information and provide consumers with reliable location-based services and information are promising and attractive research areas. Location-based services include indoor navigation and tracking, marketing (shopping advertisements, proximity-based coupon sharing), entertainment (location-based social networking, location-based gaming), location-based information retrieval (e.g., pavilion tours, underground real-time information), and emergency and safety applications (e.g., emergency calls, automotive assistance) [2,3].

While GNSS is unable to meet indoor positioning requirements due to signal attenuation and obstacles, many alternative technologies and devices are used for indoor positioning, such as Wi-Fi [4], Bluetooth [5,6], ultrasound or sound [7,8], visible light [9,10], and magnetic field [11,12]. Table 1 describes the advantages and disadvantages of these positioning techniques in detail.

Magnetic-based positioning technology has attracted continued interest in academia [13,14] and industry [15,16] due to the popularity of smartphones, tablets, and personal digital agents (PDAs) with embedded magnetometers. As an emerging indoor positioning method, magnetic-based positioning uses the Earth and the local magnetic field disturbance to achieve the goal of indoor positioning, which has the advantage of safety, reliability, low cost, and being infrastructure-free.

However, magnetic field-based positioning still has many limitations. The magnetic field intensity of the Earth’s surface varies smoothly between 23 μT and 62 μT [25]. The magnetic field measurement has only three components, which leads to its low discernibility. Embedded magnetometers of heterogeneous smartphones are produced by different suppliers with different noise tolerances and accuracies, resulting in different MF measurements of heterogeneous smartphones. Seamlessly connecting all heterogeneous smartphone positioning solutions is therefore very challenging [26].

This paper analyzes the challenges and feasibility of magnetic-based positioning through systematic experiments. In conclusion, the main contributions of this paper are as follows:

- A magnetic field acquisition system was developed using the Arduino Pro Mini and the LSM9DS1. The RLOWESS smoothing filter was proposed to eliminate the effects of noise, distortion, and outliers in the raw MF measurements.
- Static tests, trajectory tests, and rotational tests were designed to investigate the magnetic characteristics of the heterogeneous smartphone.
- Calibration tests of heterogeneous smartphones were carried out to demonstrate the potential of smartphone calibration in solving the heterogeneous device problem of MF.
- Classification tests of heterogeneous smartphones were performed to show the feasibility of magnetic field positioning.

This paper is organized as follows. In Section 2, we present related works in the literature about MF characteristics. The magnetometer measurement model is introduced in Section 3. In Section 4, we discuss the statistical characteristics of the magnetic field and its temporal stability, compare the similarity of the magnetic field trajectories of heterogeneous smartphones, and present the variation of MF intensity with device orientation for the uncalibrated case. In Section 5, we compare the characteristics of ‘calibrated’ MF for heterogeneous smartphones. In Section 6, we use different machine learning methods to compare the positioning performance of homogeneous and heterogeneous smartphones. Finally, Section 7 summarizes the paper and highlights the challenges of magnetic field positioning.

## 2. Related Work

Many research works exist in the literature relative to indoor positioning and navigation using MF measurement.

MF characteristics have been thoroughly analyzed in works [26,27,28,29,30]. Li et al. [27] conducted experiments where indoor magnetic flow density was measured in different environments. The results show stable magnetic flow density measurements over a 24 h period. The experiments were repeated three months later, and no significant change was detected. It has been observed that the MF intensity of four different smartphone models differs due to the sensors’ different sensitivities [28]. This problem can be solved by normalization, but it will reduce the accuracy of positioning. Two smartphones were also placed in the exact location for long-time data collection to demonstrate that the MF intensity is time-stable. However, due to the smartphone battery capacity limitation, the data collection time will not exceed one day. Some anomalies brought about by the change in MF over time cannot be observed. Shu et al. [30] tested MF measurements on heterogeneous smartphones with different attitudes. They showed the uniqueness of local magnetic disturbance, the temporal stability of MF measurements, and the tolerance of MF measurements to moving objects. However, they highlight that heterogeneous devices have different MF measurements in the exact location. As MF measurements are three-dimensional and directional, the reference frame of the magnetometer is not easily aligned with the world frame, which leads to the use of only the magnitude of MF measurements in practice and makes it difficult to use three-dimensional MF measurements to improve discernibility. Ashraf et al. [26] provided a comprehensive analysis of the advantages and disadvantages of MF measurement. They highlighted the heterogeneity of smartphones, the height and behavior of users, and the low discernibility of MF measurements. Smartphone-based positioning is often more complex due to complex user behavior, e.g., user height, handheld smartphone position. The work in [29] studied MF intensity and direction distribution features. Using data from accelerometers and gyroscopes to obtain rotation matrices to transform the magnetic field coordinate system will increase the system’s complexity. To avoid the coordinate transformation, the work in [14] used the gradient of the MF values between two consecutive steps to avoid calibrating different smartphones. Since the magnetic pattern from heterogeneous smartphones is not the same, the work [31] proposed to use magnetic data from multiple smartphones to make the magnetic pattern. The authors propose an algorithm that identifies outliers in the MF data, removes them, and then normalizes the selected data to calculate the magnetic pattern. The effect of user height on the performance of magnetic-based positioning algorithms with two male and two female users was analyzed in the work [32]. The MF vector measured by the magnetometer is related to the orientation of the sensor and the position and orientation can be identified by an augmented MF vector with a directional variation [33]. As the MF vector can produce many different vectors in different attitudes, it can be trained by a transformed MF vector. The calibrated smartphone and magnetometer were found to have similar magnetic fingerprints [34]. GPS and Wi-Fi, as well as hand-held smartphones, do not significantly affect the measurement results. The peaks and drops of the MF signal can be used to identify certain areas. The effects of different metals and electronic objects on the MF were compared. MF measurements in indoor environments obviously vary more than those in outdoor environments. The quality of the positioning depends on the number of components used. When all three components are used, the performance of MF positioning is good. When using only the magnitude, or the vertical and horizontal components, the positioning performance decreases rapidly.

Indoor mapping is a prerequisite for indoor positioning systems. He et al. [30] use a site survey method based on compliance walks. This traditional map construction requires hiring experts to build indoor maps and update them regularly, which is an expensive and time-consuming method that cannot be applied to large-scale indoor coverage. In recent years, crowdsourcing, SLAM, as well as Gaussian processes have been proposed for constructing magnetic maps [35]. Crowdsourcing is an approach that uses crowd contributions to achieve complex tasks and is well suited for magnetic map construction [36]. For example, the works [37,38,39] used crowdsourcing to merge sensor data from multiple users on different paths to build a magnetic map. PFSurvey [40] uses accelerometer, gyroscope, and magnetometer data to estimate the trajectory of the surveyor and uses SLAM and particle filters to incorporate the floor plans of buildings, allowing for the rapid construction of magnetic field maps. PFSurvey’s data collection costs are low, yet it achieves similar accuracy to traditional site survey methods. Combining the physical properties of the magnetic field with a Gaussian process to model the magnetic field components allows for continuous updating of the estimates and the time variation of the magnetic field to build magnetic field maps quickly [41,42,43,44,45]

Magnetic field positioning methods can be divided into five categories: magnetic landmark, dynamic time warping, filtering method, SLAM, and neural network method.

Equipment with ferromagnetic materials (e.g., refrigerators, lifts, metal doors, etc.) can cause MF measurements to show prominent variations, and this magnetic disturbance can be used as a magnetic landmark to enhance indoor positioning and mapping [46,47,48], to detect indoor/outdoor environments [49], and to label the semantics of indoor environments [50].

Different walking speeds and sampling rates produce different numbers of magnetic samples within the same spatial coverage. Dynamic time warping (DTW) could align two magnetic field sequences with similar patterns but different amplitudes and times by compressing or stretching the time axis of one (or both) magnetic field sequences. Subbu et al. [51] use DTW to classify magnetic signatures collected from different corridors. By aligning unknown magnetic signatures with known signatures, the technique can obtain a close match between test and specific, temporally distinct magnetic signatures, thus providing correct corridor/location information. Through a similar method, Perez-Navarro et al. [52] showed that magnetic fields are a promising positioning mechanism when only the user’s zone needs to be located. Wang et al. [53] used DTW as a distance function to quantify the similarity between two signature vectors with different spatial sampling densities to match magnetic trajectories. The matched magnetic trajectories were used to calibrate the pedestrian dead reckoning (PDR) position, and the authors used online-collected magnetic trajectories to construct backward magnetic trajectories to increase the probability of improving the PDR position. By averaging the direction of travel for all steps between two successive detected turns, the authors reduced fluctuations in the direction of travel caused by user movement, sensor noise, local magnetic anomalies, and other electrical disturbances. In addition, 3DDTW [54], which extends the one-dimensional input signal into a two-dimensional signal, calculates the distance between the MF measurement and the magnetic fingerprinting, thus reducing the mismatch between the magnetic signature. CSMS [55] integrates channel state information (CSI) and magnetic field strength (MFS). Initial positions are first obtained by the M-KNN method. Then, DTW is applied to match the magnetic sequence during the motion for tracking. Finally, the k-nearest neighbor algorithm calculates the weights and narrows down the positioning range for fingerprint matching.

Machine learning methods such as K-NN [56], support vector machines (SVM) [57], naive Bayes [58], decision trees [59], and discriminant analysis [60] are widely used to extract the core features of a signal. The advantage of the machine learning approach is its ability to learn helpful information from input data with known or unknown statistics [61]. Galván-Tejada et al. [62] proposed a "signature" obtained by walking randomly around the room, using the spectrum, skewness, kurtosis, and variance of the magnetic signal as features. A genetic algorithm (GA) was used as the feature selection algorithm for data dimensionality reduction. However, the authors used only one-dimensional MF intensity and did not consider calibrating MF measurements of heterogeneous smartphones. Chuang et al. [11] designed a system with four magnetometers to evaluate the MF strength in each direction and used the nearest neighbor method to classify MF measurements with 12 components. The MF measurements of two different floors could be distinguished. The effects of smartphones, watches, laptops, elevators, and workbenches on the intensity of the MF were analyzed. Their system used chest-hung magnetometers instead of smartphones, which, in reality, do not have four magnetometers to measure the magnetic fingerprints in all directions.

Filter-based methods (e.g., Hidden Markov Model (HMM), Kalman filter, particle filter) have many indoor magnetic field positioning applications. Filter-based methods can fuse data from multiple sensors to provide higher positioning accuracy. For example, the work of Basmag [63] used a Backward Sequence Matching Algorithm (BSMA) to optimize the HMM and improve the low discriminability of the magnetic signal with the help of PDR. An HMM-based unsupervised learning algorithm was proposed in work [64] to compare the similarity of magnetic fingerprints with a lightweight algorithm. The extended Kalman filter could reduce the cumulative error of inertial sensors and improve orientation and positioning accuracy [65]. A reliability-augmented particle filter was mentioned in [66]; they used a dynamic step estimation algorithm and a heuristic particle resampling algorithm to reduce the error of motion estimation and improve the robustness of the particle filter. The work [67] employed the extended Kalman filter and particle filter to fuse information obtained from pedestrian dead reckoning and magnetic fingerprints, showing higher positioning performance than using the particle filter alone.

SLAM-based magnetic field positioning was recently presented in the work [47,68]. MagSLAM [68] used MF and human stride measurements without using a priori maps. Unique patterns in a smartphone’s accelerometer when climbing stairs, unusual magnetic disturbance at specific locations, and unique Wi-Fi access can serve as unique signatures in indoor environments. SemanticSLAM [47] adopted these unique signatures as landmarks and combined them with the pedestrian dead reckoning in the SLAM framework to reduce localization errors and convergence times. This approach is proven to be calibration-free. SLAM based on magnetic field measurements has two main challenges. Firstly, the construction of maps for large-scale indoor environments is challenging. Secondly, the continuous data exchange between the map and the positioning algorithm is energy-intensive [69].

Artificial neural networks (ANNs) are often used for classification and prediction, and researchers have recently applied ANNs to MF positioning. The MF maps are used to train the NN in the offline phase. The real-time MF measurements are fed into the trained NN to estimate its position in the online phase. The two main types of ANN-based magnetic field positioning are convolutional neural networks (CNN) and recurrent neural networks (RNN). The work in [32,70,71,72,73] uses CNNs to convert MF fingerprints into “image patterns” for classification, and the work in [74,75,76,77,78] uses RNNs to predict MF measurements as time series.

There are several limitations to the above studies. Firstly, they directly used the magnetic field data to predict the user’s current location, without pre-processing the magnetic field in conjunction with the magnetometer measurement model. Secondly, due to the battery capacity limitations of smartphones, magnetic field measurements cannot be collected continuously for long periods. Thirdly, although several research works have given solutions for heterogeneous smartphones, it is not clear whether the calibration parameters will change over time and whether they need to be calibrated at every point in space. Fourthly, uncalibrated magnetic field measurement varies with the orientation of the device. Magnetic field measurements in a specific direction can only be matched to the magnetic field fingerprint in that specific direction. Finally, the above studies used homogeneous smartphones and did not use fingerprint databases from heterogeneous smartphones when testing the positioning algorithms. This study aims to provide an exhaustive analysis of magnetic field-based indoor positioning and experimentally explore the feasibility and challenges of magnetic field-based indoor positioning.

## 3. Magnetometer Measurement Model

The magnetometer measures the MF intensity along the *x*, *y*, and *z* axis of the sensor. Magnetometers are essential auxiliary sensors for attitude estimation in low-cost, high-performance inertial navigation systems. The fusion of magnetometer and inertial sensor can obtain accurate 3D attitude estimation [79,80]. The accuracy of 3D attitude estimation is closely related to the calibration of sensor measurements [81]. Magnetometers are more sensitive to environmental changes than inertial sensors and require frequent recalibration [82]. Generally, there are two main types of error sources in magnetometers. One type is the non-orthogonality, scale factor, and bias caused by technical limitations in the sensor’s manufacturing, installation, and materials. The other is the hard and soft iron effect caused by ferromagnets around the sensor [83].

Complete three-axis magnetometer measurements can be expressed as:

(1) $$m^{b}=\mathrm{SM}\left(A_{\mathrm{si}}R^{\mathrm{bn}}m^{n}+b_{h_{i}}\right)+b_{\mathrm{so}}+\varepsilon .$$

In Equation (1), $m^{n}\in R^{3\times 1}$ represents the Earth’s geomagnetic field vector in navigation frame *n* aligned with the Earth’s gravity. $m^{b}\in R^{3\times 1}$ represents the measured magnetic vector in the sensor body frame *b*. $R^{\mathrm{bn}}\in R^{3\times 3}$ represents the rotation matrix that transforms the magnetic vector from the navigation frame *n* to the sensor body frame *b*. The scale factor $S\in R^{3\times 3}$ represents the difference in sensitivity of the three axes. The matrix $M\in R^{3\times 3}$ indicates the misalignment errors of sensors. The vector $b_{\mathrm{so}}\in R^{3\times 1}$ represents the bias in sensors. The matrix $A_{\mathrm{si}}\in R^{3\times 3}$ represents the soft iron distortion. The vector $b_{\mathrm{hi}}\in R^{3\times 3}$ represents the hard iron bias. ε represents an i.i.d Gaussian noise from $N\left(0,\sigma_{\varepsilon }^{2}I\right)$. Each of these error terms will be discussed in detail below.

Suppose that $\varepsilon_{x}\in R^{3\times 1},\varepsilon_{y}\in R^{3\times 1},$ and $\varepsilon_{z}\in R^{3\times 1}$ represent the skew error of sensors’ *x*, *y*, and *z* axis in the sensor frame, respectively. M can be modeled as:

(2) $$\begin{array}{c} M={\left[\begin{array}{ccc} \varepsilon_{x} & \varepsilon_{y} & \varepsilon_{z} \end{array}\right]}^{-1}. \end{array}$$

The scale factor errors are modeled as

(3) $$\begin{array}{c} S=\mathrm{diag}\left(\left[\begin{array}{ccc} s_{x} & s_{y} & s_{z} \end{array}\right]\right), \end{array}$$

where $s_{x}$, $s_{y}$, and $s_{z}$ denote the scale factor of the sensors’ *x*, *y*, and *z* axis, respectively.

The sensor bias is modeled as

(4) $$\begin{array}{c} b_{\mathrm{so}}={\left[\begin{array}{ccc} b_{{\mathrm{so}}_{z}} & b_{{\mathrm{so}}_{y}} & b_{{\mathrm{so}}_{z}} \end{array}\right]}^{⊤}, \end{array}$$

where ${\mathrm{so}}_{x}$, ${\mathrm{so}}_{y}$, and ${\mathrm{so}}_{z}$ denote the sensor bias of the sensors’ *x*, *y*, and *z* axis.

In an outdoor environment, the local geomagnetic field is equal to the local Earth’s MF. Its horizontal component points to the magnetic north pole of the Earth [84]. In the absence of any magnetic interference, by rotating the magnetometer in all possible directions, the MF measurements are located on a sphere with a radius equal to the MF intensity.

**Figure 1:**
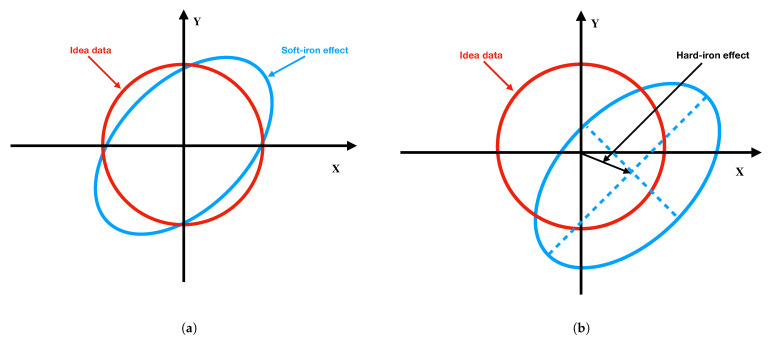
Soft and hard iron effects: (**a**) soft iron effect; (**b**) hard iron effect.

However, metals such as nickel and iron could cause the soft iron effect, which distorts the sphere into an ellipsoid, as shown on the plot of Figure 1a. This distortion is denoted by $A_{\mathrm{si}}\in R^{3\times 3}$. The hard iron effect is denoted $b_{\mathrm{hi}}\in R^{3\times 3}$ and is produced by materials that exhibit a constant additional field to the Earth’s MF, shown in Figure 1b. This distortion shifts the origin of the ideal magnetic measurement sphere. Hence, with the hard iron and soft iron effect, the magnetometer measurement model can be written as:

(5) $$m^{b}=A_{\mathrm{si}}R^{\mathrm{bn}}m^{n}+b_{\mathrm{hi}}.$$

It is not necessary to identify all the components of Equation (1). Expanding Equation (1), the scale factor, misalignment, and soft iron distortion can be combined into a distortion matrix $A\in R^{3\times 3}$, and the hard iron effect and sensor bias can be formed into an offset vector $b\in R^{3\times 1}$.

(6) $$\begin{array}{cc} A & =SMA_{\mathrm{si}}, \end{array}$$

(7) $$\begin{array}{cc} b & =SMb_{\mathrm{hi}}+b_{\mathrm{so}}. \end{array}$$

Hence, the magnetometer measurement model can be expressed as:

(8) $$\begin{array}{c} m^{b}=AR^{\mathrm{bn}}m^{n}+b+\varepsilon . \end{array}$$

In the following section, we will use the magnetometer measurement model to analyze the MF signal, the statistical characteristics of a heterogeneous device, and the influence of the distortion matrix A and the offset b.

## 4. Analysis of the MF Characterisctics

In this section, we analyze the MF measurement characteristics of a heterogeneous smartphone through static, trajectory, and rotation tests. Several heterogeneous smartphones were used in the experiments. Table 2 describes the system version, the sensor vendors, the model, and the characteristics of the magnetometer. Android sensor information is available through the Android API, while iPhone sensor information is not available directly from the iOS API but only through publicly available information. The Huawei P9, Redmi Note 10 Pro, and Samsung S9 all use different magnetometer models from AKM (Asahi Kasei Microdevices), and Bluebird uses magnetometers from MEMSIC Inc. As the iPhone has no interface to obtain detailed information about the sensors [85,86], we could not find information on the iPhone sensor’s manufacturer, model, range, and resolution. The heterogeneity of smartphones is caused by the different characteristics of magnetometers, making it very difficult to design a positioning system that can seamlessly connect various smartphones [26,32].

This paper investigates the MF measurements of the Android and iOS devices. All smartphones used MATLAB Mobile software to collect MF measurements to prevent the effects of software differences. In Section 4.1, we study the statistical characteristics and Gaussianity of heterogeneous smartphones. In Section 4.2, we compare the MF trajectories of heterogeneous smartphones, and in Section 4.3, we test the MF characteristics of smartphones when they are rotated. In Section 4.4, we design a magnetic field acquisition device using the Arduino Pro Mini and the LSM9DS1 and analyze the original magnetometer’s statistical characteristics.

### 4.1. Statistical Tests with Heterogeneous Smartphones

Ferromagnetic materials in modern buildings, such as steel casing, electrical equipment, vending machines, etc., can cause local magnetic disturbance. Magnetic-based indoor positioning requires that the magnetic field measurement remain stable over a relatively long time. It is important to design a positioning method that can seamlessly integrate with the MF measurements of various smartphones [26]. Hence, we first investigate the indoor magnetic field’s temporal stability, Gaussianity, and heterogeneity.

Three heterogeneous smartphones (iPhone Xs Max, Huawei P9, Bluebird) were used to investigate the MF’s statistical characteristics with heterogeneous smartphones.

The three smartphones were placed at a specific point on a wooden table without ferromagnetic material, and the orientation was kept consistent, with a sampling frequency of 100 Hz during a period of 35 min. We found that although the experiment was set at a sampling frequency of 100 Hz, the heterogeneous smartphones could not always reach the set frequency. The iPhone Xs Max sampled at 100 Hz, the Huawei P9 at 103 Hz, and the Bluebird could only reach 80 Hz. The sampling frequency that we can achieve is related to the processor performance of the smartphone.

**Figure 2:**
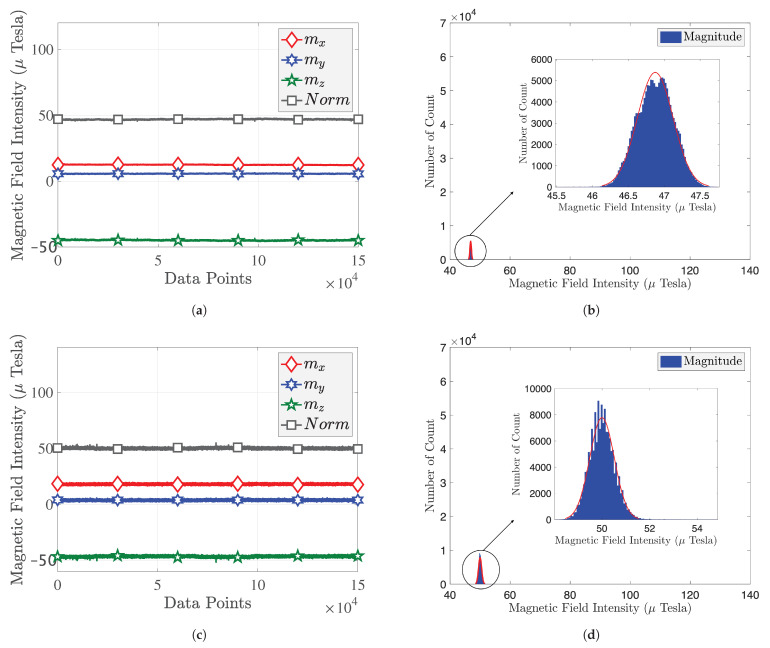

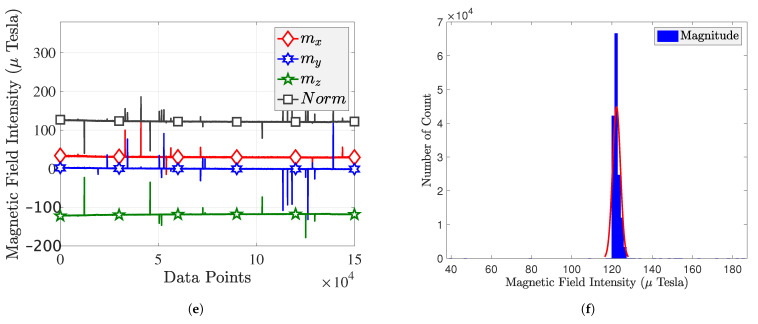
MF measurement of heterogeneous smartphones: (**a**,**c**,**e**) are the MFs of iPhone Xs Max, Huawei P9, and Bluebird, respectively. (**b**,**d**,**f**) are the histogram of magnitude for iPhone Xs Max, Huawei P9, and Bluebird, respectively.

Figure 2a,c,e demonstrate the temporal stability of the MF measurements of heterogeneous smartphones at a specific location. The MF reading of the iPhone Xs Max and Huawei P9 is quite stable (at least, during a short observation time period), while the MF measurements of the Bluebird are corrupted by outliers and strongly biased compared to the other two groups. From Figure 2b,d, we can see that the MF magnitude follows approximately a normal distribution. However, Figure 2f shows that Bluebird’s MF measurements do not follow a normal distribution due to the effect of outliers.

Table 3 summarizes the mean values, standard deviations, kurtosis, and skewness of MF intensity with heterogeneous smartphones. From the column of standard deviation, the standard deviation of the iPhone Xs Max is relatively low. Huawei’s standard deviation is moderate, while Bluebird’s standard deviation is the largest and the measurement uncertainty is the highest. Usually, the smaller the standard deviation of the smartphone, the smaller the fluctuation of the MF signal, the higher the certainty of MF measurement, and the better the positioning performance.

From the column of mean values, the MF intensity of the iPhone and Huawei are at the same level, while Bluebird’s measurements are biased, well outside of the geomagnetic range of 23 μT to 62 μT [87]. The magnetometer calibration algorithm based on ellipsoidal fitting can correct the measurement distortions. The iPhone’s kurtosis and skewness show a right-skewed normal distribution. At the same time, the Bluebird’s measurements have many outliers, resulting in much larger kurtosis and skewness than those of the iPhone and Huawei. Based on the above analysis, we found that different smartphone manufacturers have different embedded magnetometer models, resulting in different MF measurements. Moreover, differences in processor performance make it difficult to achieve consistent sampling frequencies across heterogeneous smartphones.

### 4.2. Trajectory Test with Heterogeneous Smartphone

From Section 4.1, we have already confirmed that MF does not change over a short time period. In this section, we study the spatial distribution variability and temporal stability of heterogeneous smartphones’ MF measurement. Users walked along the same path with heterogeneous smartphones to collect data at a sampling frequency of 100 Hz.

The experiment took place on the third floor of Polytech Galilee (Orléans, France), and the tested heterogeneous smartphones included the iPhone Xs Max, Samsung S9, Redmi Note 10 Pro, and Huawei P9.

**Figure 3:**
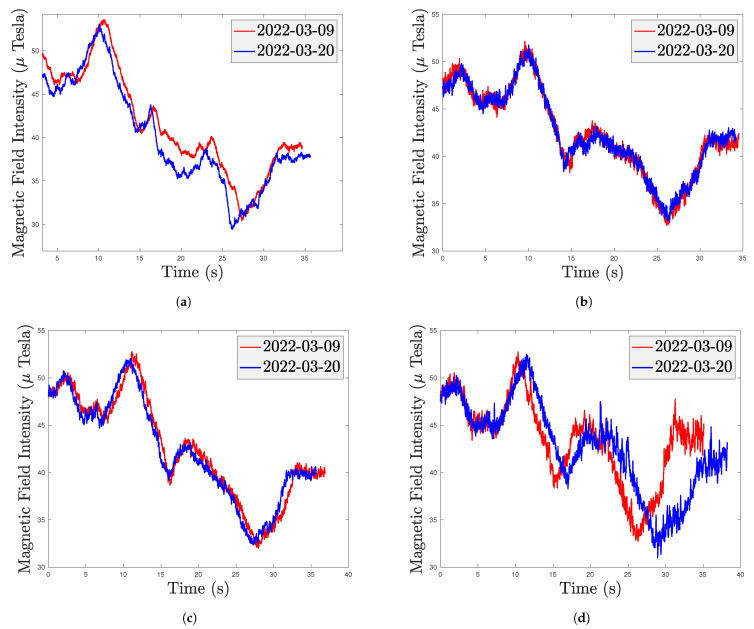
MF measurements of heterogeneous smartphones at different dates on the same path. (**a**) iPhone Xs Max; (**b**) Samsung S9; (**c**) Redmi Note 10 Pro; (**d**) Huawei P9.

**Figure 4:**
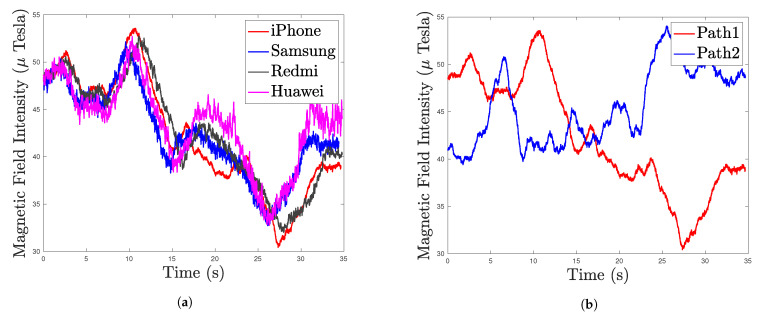
Trajectory test: (**a**) Comparison of MF measurements of heterogeneous smartphones in the same path. (**b**) Comparison of the iPhone Xs Max’s MF measurements under two different paths.

As shown in Figure 3, the trajectories of the heterogeneous smartphones tested at different dates largely overlap, demonstrating that the indoor MF is stable over time without significant changes to the indoor infrastructure. The magnetic fingerprint, including local indoor disturbances, is stable over time without changes in indoor infrastructure. Previous works [11,26,27,30,88,89] have preliminarily explored these properties of MFs. Figure 4a shows that the heterogeneous smartphones have similar MF measurement trajectories under the same path.

Disturbances caused by building materials and electrical appliances lead to certain anomalies in the indoor MF. The pattern of disturbances varies from place to place, making the indoor MF spatially unique. Figure 4b shows that the smartphone has different MF measurements under different paths, demonstrating the MF’s spatial uniqueness.

### 4.3. Rotation Test

Magnetometers are often fused with inertial sensors for pose estimation. Accurate MF measurements are essential for determining the user’s heading and orientation. The construction of an MF map would be a tedious task if the MF measurement was dependent on orientation. MF measurements in various orientations at a specific point have been investigated.

**Figure 5:**
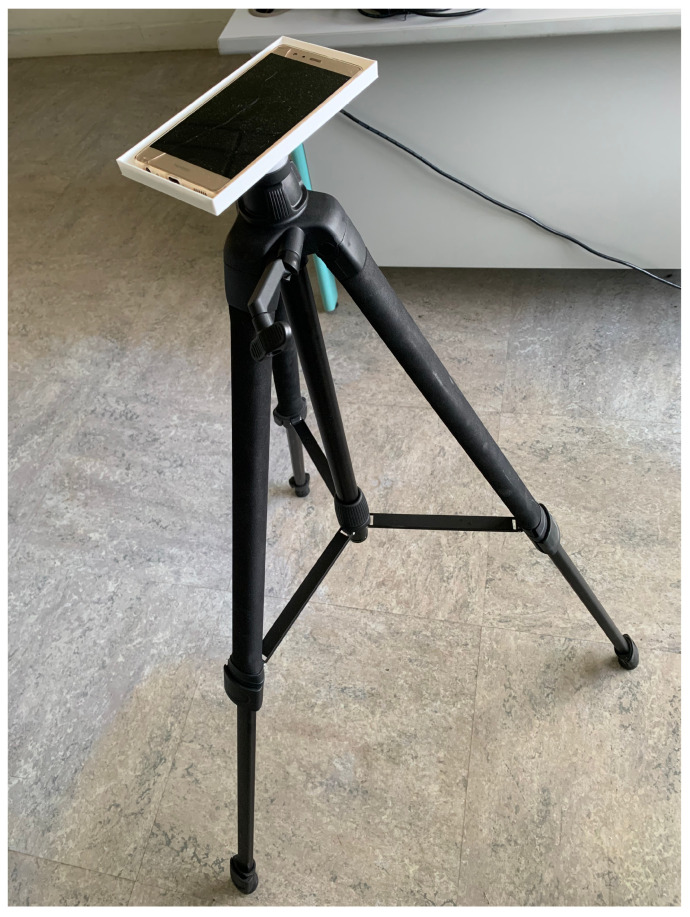
Rotatable and height-adjustable platform.

A smartphone was placed on a rotatable platform, as shown in Figure 5; the smartphone frame was aligned with the platform frame, and the platform was rotated around the z axis of the smartphone. The MF database was collected at a sampling frequency of 100 Hz for 25 s.

**Figure 6:**
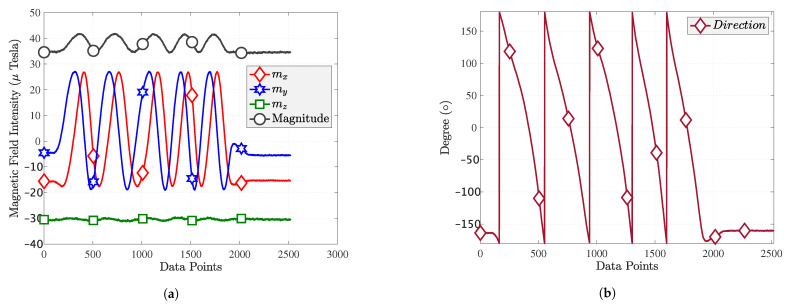
Smartphone rotation test: (**a**) magnetic field with rotation; (**b**) magnetic direction.

MF positioning is based on the stability and uniqueness of the MF signature. In the positioning phase, smartphone measurement should match the MF database. The location with the shortest distance has the highest probability. However, as seen in Figure 6a, the *x*, *y* component and magnitude of MF also change periodically when the smartphone rotates periodically; only the *z* components remain relatively stable. Instead of using MF magnitude as a signature, it is better to use the *z* component as a fingerprint.

The *x* and *y* axis of the MF can represent the variation in the magnetic direction. Figure 6b shows that the magnetic orientation varies between $-180^{\circ }$ and $180^{\circ }$, where the magnetic direction is calculated as follows:

(9) $$\begin{array}{c} Orientation=\arctan\left(\frac{m_{y}}{m_{x}}\right). \end{array}$$

The ideal magnetic direction should be a standard sine curve, but the curve shown in Figure 6b is clearly not a sine curve and does not accurately represent the magnetic direction.

**Figure 7:**
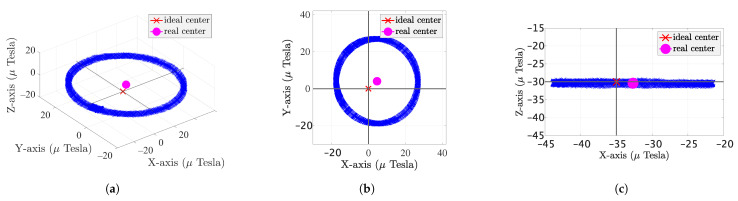
Ellipse plot: (**a**) xyz plot; (**b**) xy plot; (**c**) xz plot.

Figure 7a shows the MF measurements on the *x*, *y*, and *z* axis, where the MF measurements are approximately circular, illustrating that the soft iron effect can be ignored. Figure 7b shows the projection of the MF measurement on the *xy* coordinate; the actual center of the circle is at (4.65,4.01) instead of at the ideal origin (0,0), which indicates a hard iron effect of the MF measurement. Therefore, calibration of the smartphone is necessary. Figure 7c shows the projection of the MF measurement in the *xz* coordinate. The results show that the magnetic field does not change on the *z* axis, and the offset originates from the *x* and *y* axis.

### 4.4. Static Tests with Magnetometer

**Figure 8:**
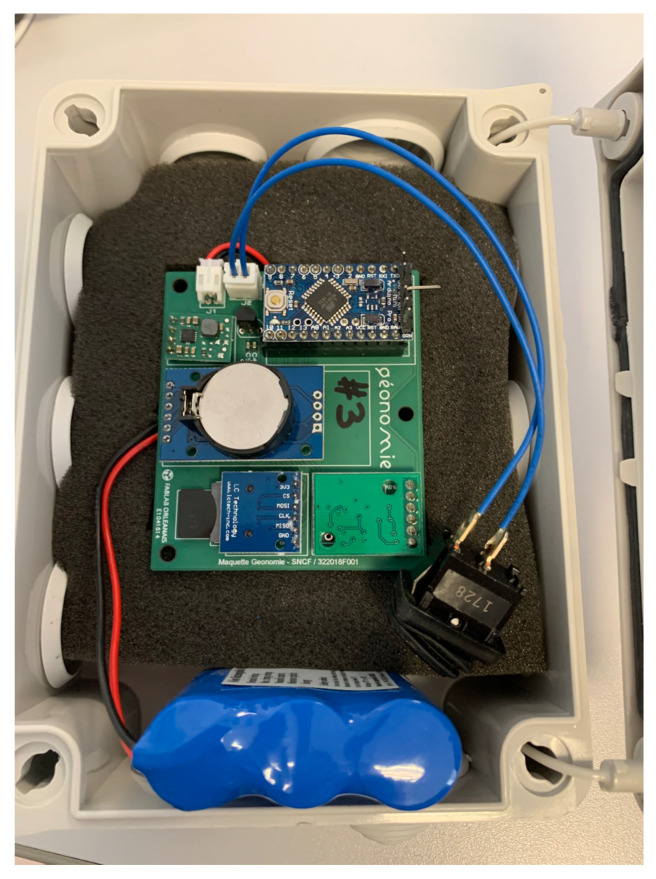
Nine-DoF LSM9DS1 embedded with Arduino Pro Mini.

The previous analysis used a commercial smartphone with already pre-processed MF measurements by the manufacturer. An MF acquisition device, shown in Figure 8, was designed with the Arduino Pro Mini and 9-DoF LSM9DS1 [90] to study the unprocessed MF measurements; the software was developed using the open-source code available on the Arduino website [91]. Six designed devices (referred to as D1, D2, D3, D4, D5, and D6) were placed in a warehouse of width 45.69 m and height 49.5 m in the city of Tour, France. They continuously collected magnetic measurements in stationary mode for 9 days until the battery ran out.

The robust locally weighted scatterplot smoothing (RLOWESS) method has been applied for outlier rejection [92]. Indeed, robust estimation methods generally involve the detection and mitigation of outliers, e.g., [31]. In our context, this pre-processing step is to avoid the large positioning bias that might be induced by such outliers. The robust weightings, multiplied with the neighborhood weight, are used for re-estimating a linear regression function:

(10) $$\begin{array}{c} \sum_{i}w_{i}\eta_{i}{\left(r_{i}\right)}^{2}, \end{array}$$

where $w_{i}$ is a neighborhood weight expressed as:

(11) $$\begin{array}{c} w_{i}={\left(1-{\left|\frac{x-x_{i}}{{˘}_{q}\left(x\right)}\right|}^{3}\right)}^{3}, \end{array}$$

x is the predictor, $x_{i}$ is the nearest neighbor of x as defined by the window slide, and ${˘}_{q}\left(x\right)$ is the distance of the *q*th farthest $x_{i}$ from x within the window slide.

$\eta_{i}$ is the robust weight expressed as a bisquare function:

(12) $$\begin{array}{c} \eta_{i}=\left\{\begin{array}{cc} {\left(1-{\left(r_{i}/6h\right)}^{2}\right)}^{2}, & |r_{i}|<6h\\ 0, & |r_{i}|\geq 6h \end{array}\right. \end{array}$$

where $r_{i}$ is the residual of the response value y and the predicted response value $\hat{y}$, and h is the median of the residuals

(13) $$\begin{array}{c} h=\mathrm{median}(|r_{i}\left|\right)=\mathrm{median}\left(\left|y_{i}-{\hat{y}}_{i}\right|\right). \end{array}$$

**Figure 9:**
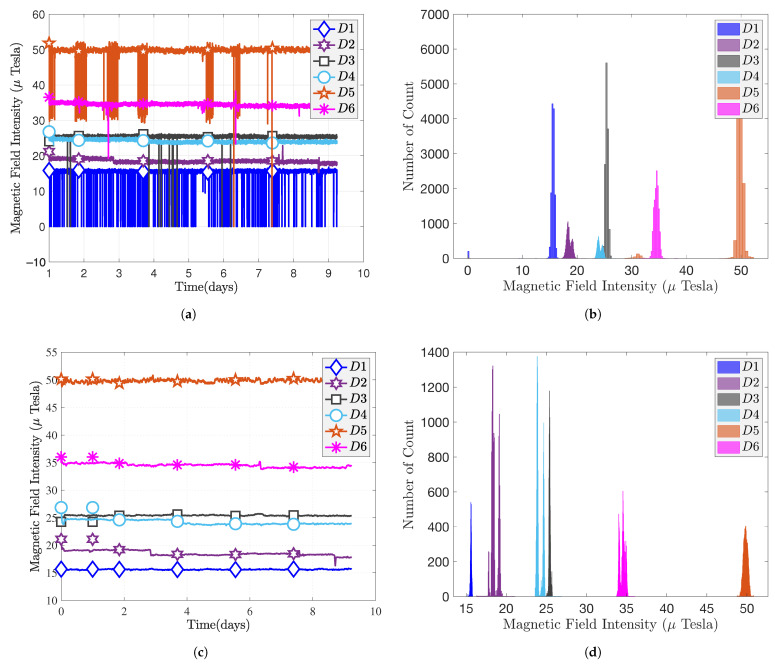
Nine-DoF LSM9DS1 sensor’s measurements: (**a**) original magnitude; (**b**) original magnitude histogram; (**c**) smoothing magnitude; (**d**) smoothing magnitude histogram.

In the following, we use the RLOWESS algorithm to smooth the MF measurements. Figure 9a shows that magnetic fingerprinting is not stable before filtering. There will be a sudden jitter without a calibration step.

Figure 9b provides the histogram of magnetic measurements before processing, We can see that there exist spurious values due to the jitters for D1, D3, D5, and D6, i.e., D1 has some values of 0 to 15 μT, while D5 has some values in the range of 30 to 50 μT. A similar situation has been found for the Bluebird, where we believe that smartphone manufacturers have different standards for the calibration of magnetometers, resulting in different MF measurements. In this experiment, we used the RLOWESS method mentioned above to filter out outliers of MF measurement. The filtering result is shown in Figure 9c, and the histogram of filtered MF is given in Figure 9d. As we can see, the magnitude of MF intensities is different at different positions. The six quasi-normal distributions could be distinguished by visual inspection in this particular case. We can draw the conclusion from Table 4 that MF is stable for the considered period of time and subject to quasi-normal distribution. However, the conditioning of the sensors (or pre-filtering of their outputs) is necessary since all measurements are affected by outliers, which results in very high-level variance. The higher the variance, the worse the performance for positioning.

## 5. Calibration of Magnetic Field

Magnetometer calibration of a smartphone is actually the estimation of the calibration parameters A and b in Equation (8). In this paper, we use the batch magnetometer calibration method, where the smartphone is rotated along each direction to obtain magnetic field data, using the entire set of magnetic field measurements to estimate the unknown calibration parameters [93]. Supposing that magnetometer measurements are constant, and the local MF measurements are attitude-independent, to estimate the magnetometer error term, we can construct a cost function from the difference between the magnetometer measurement model and the true MF measurements [94]. Here, we present a calibration test with a different smartphone, and the calibration algorithm uses the work in [95]. We calculate the soft iron A and hard iron b of the smartphone at a given point and a given date. We tested these transformations (i.e., A and b) over a period of time to see if periodic re-calibration is necessary. Moreover, we tested the same calibration parameters at different points to see if they depend on the ambient location or only on the considered smartphone. To accomplish these goals, three smartphones (iPhone Xs Max, Huawei P9, Bluebird) have been used to continuously collect MF data at 10 points from February to June 2020 in 2 buildings; point 1 to point 7 are in building 1; point 8 to point 10 are in building 2. Smartphones were rotated around X, Y, Z, respectively, for 120 s for the aim of calibration.

**Figure 10:**
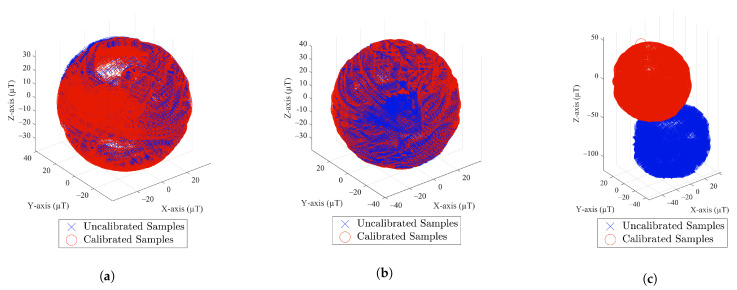
Smartphone calibration test: (**a**) iPhone Xs Max; (**b**) Huawei P9; (**c**) Bluebird.

Figure 10 shows the calibration results at P1 on 7 February 2022, where the MF measurements of these smartphones are found to be spherical. This means that their distortion matrix approximates the identity matrix and the bias is negligible. Meanwhile, there is a significant bias for Bluebird (shown in Figure 10c), which needs to be eliminated.

We calculated calibration parameters using MF measurements of P1 from three smartphones on 7 February 2020. The obtained calibration parameters were applied to Equation (14) to obtain true MF measurements at different times.

(14) $$\begin{array}{c} m^{n}=R^{\mathrm{nb}}A^{-1}m^{b}-b-\varepsilon . \end{array}$$

**Figure 11:**
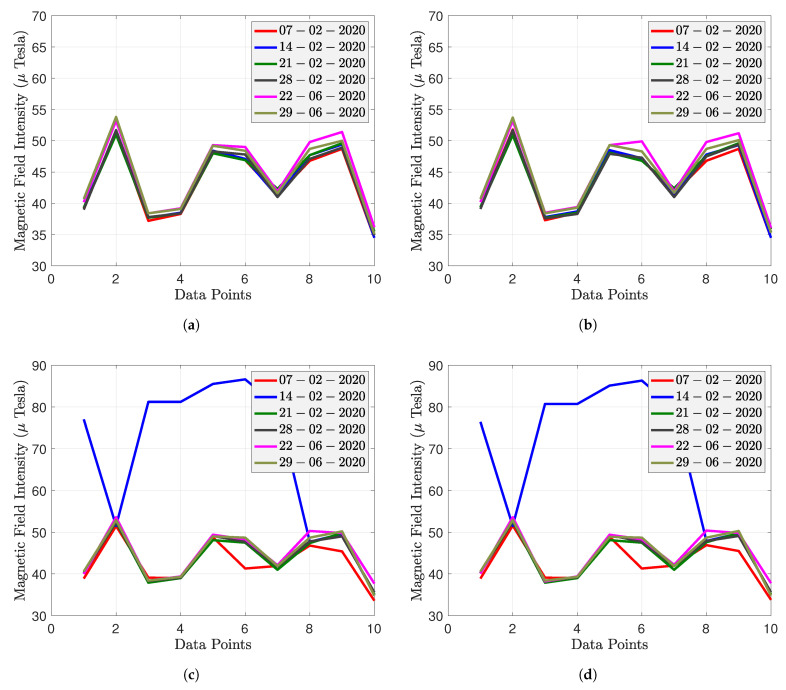

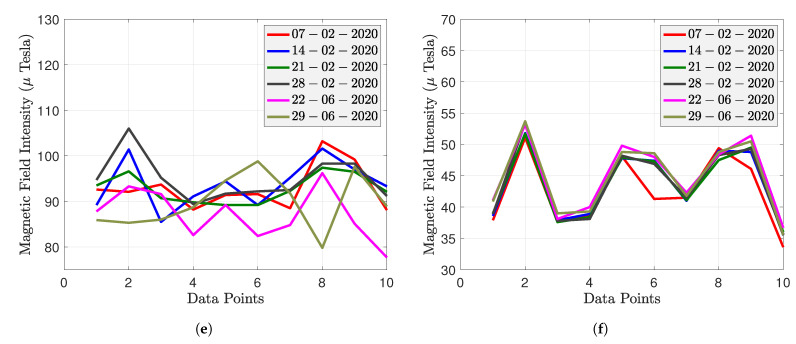
Magnitude of heterogeneous smartphones from 7 February 2020 to 29 June 2020: (**a**,**c**,**e**) are the original MF magnitudes of iPhone Xs Max, Huawei P9, and Bluebird, respectively. (**b**,**d**,**f**) are the calibrated MF magnitudes of iPhone Xs Max, Huawei P9, and Bluebird, respectively.

The rotation matrix and bias obtained at point P1 on the first day were used to calibrate all other data to demonstrate that the rotation matrix and bias of the MF are independent of time and space and only relate to the internal parameters of the smartphone. Figure 11a,c,e show the original MF magnitude of the iPhone Xs Max, Huawei P9, and Bluebird. Figure 11b,d,f show the calibrated MF magnitude of the iPhone Xs Max, Huawei P9, and Bluebird, respectively.

The X axis represents the 10 points where the magnetic field was collected. When we connect the MF intensity of the 10 points, we find that the six datasets collected over five months overlap, proving the magnetic field’s temporal stability. MF intensity is different for P1 to P10, indicating that the MF measurement has spatial discernibility.

As shown in Figure 11c, Huawei’s MF measurements from P1 to P7 on 14 February 2020 are abnormal (blue line), but the rest of the time, the MF measurements follow the same trend as the iPhone Xs Max.

Figure 11e shows that the Bluebird’s original MF measurements do not overlap. The MF intensity varies between 80 and 110 μTesla, much greater than the iPhone Xs Max and Huawei P9. However, the calibrated MF intensity (shown in Figure 11f) is essentially the same as the MF intensity of the iPhone Xs Max and Huawei P9, suggesting that magnetometer calibration can solve the problem of smartphone heterogeneity.

**Figure 12:**
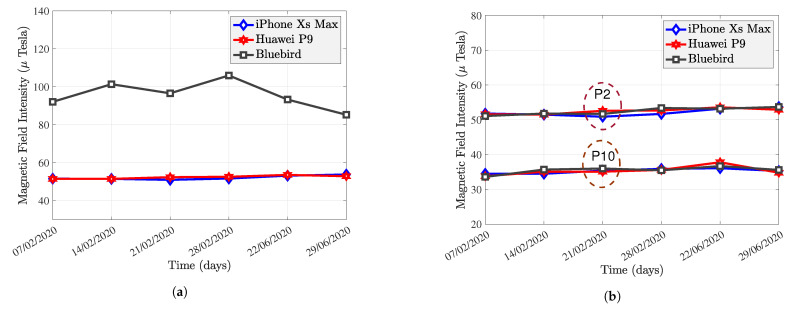
Comparison of heterogeneous smartphones. (**a**) Uncalibrated MF measurement of P2. (**b**) Calibration result of P2 and P10.

Figure 12a compares the MF intensity of the three smartphones from February to June. The MF intensities of the iPhone and Huawei are stable and overlap. Assuming that the iPhone is used to construct an MF map, Huawei can also use this MF map for positioning. However, the MF intensity of Bluebird is significantly different from the other two devices. The difference in the MF measurements of the heterogeneous smartphone is a significant challenge for MF positioning.

Figure 12b compares the calibrated magnitude of the P2 and P10 from February 2020 to June 2020. The calibrated MF intensity varies within a relatively stable range over five months, indicating that the calibration transformation is “stable” over time and over space. Heterogeneous smartphones have similar MF measurements at the same points. One can conclude that the MF calibration parameters are determined by the characteristics of the smartphone, and not by the environment. In addition, magnetometer calibration can solve the problem of heterogeneous equipment for MF.

Based on the above analysis, the original and calibrated magnitude of MF at each point remains stable over five months, while the surrounding environment remains unchanged. Different points have different mean values of MF intensity, which is an advantage for magnetic fingerprinting positioning. For the Huawei P9 and iPhone Xs, they have already been initially calibrated by the smartphone manufacturer. The iPhone Xs Max has the slightest standard deviation in MF intensity of the three smartphones. On 14 February 2020, the Huawei P9 had anomalies at P1, P3, P4, P5, P6, and P7. The standard deviation of these points is so significant that calibrating these values using the rotation matrix and bias from P1 on the first day would have resulted in substantial errors. The original magnitude of the Bluebird is not at the same level as the iPhone Xs Max and Huawei P9. However, the calibrated magnitude is at the same level for all three smartphones.

## 6. Classification Test with Calibration

The rapid development of artificial intelligence has attracted the attention of researchers who are applying machine learning methods to indoor positioning technology to address the limitations of traditional positioning techniques. The most important advantage of the machine learning approach is its ability to learn helpful information from input data with known or unknown statistics [61].

Classifier algorithms such as k-NN [56], support vector machines (SVM) [57], naive Bayes [58], decision trees [59], and discriminant analysis [60] are widely used to extract the core features of a signal in machine-learning-based positioning.

Positioning methods often need to consider positioning accuracy and computational complexity. Obtaining high-dimensional data through feature engineering can improve accuracy, but it also brings higher computational complexity. Dimensionality reduction techniques such as principal component analysis (PCA) [96] and singular value decomposition (SVD) [97] can transform high-dimensional features into low-dimensional features, significantly reducing the storage space and computational complexity of magnetic fingerprint-based positioning.

**Figure 13:**
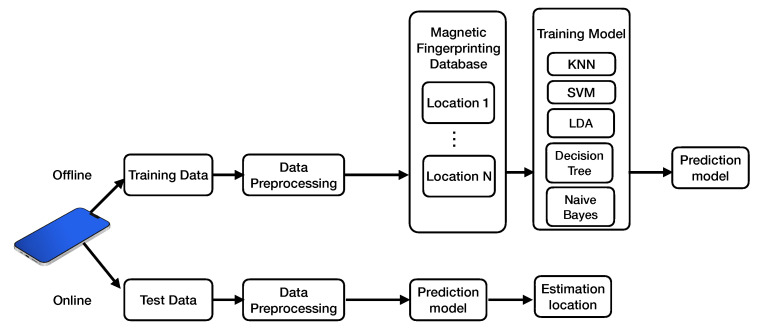
Architecture of magnetic-based positioning system.

The architecture of a magnetic-based positioning system is shown in Figure 13. The system consists of two phases: online and offline [24]. First, raw magnetic data are collected at a reference location using a smartphone. Then, during the data pre-processing step, low-pass filters, smoothing filters, and calibration algorithms are applied to remove noise and bias from the smartphone’s MF measurements. Thirdly, the pre-processed data construct a fingerprinting database corresponding to each location. The fingerprinting database is trained using machine learning methods (e.g., kNN, support vector machines, decision trees, naive Bayes, discriminant analysis) to obtain predictive models. Finally, a prediction model is used to predict the test data’s position. We will focus on fuzzy kNN, support vector machines, decision trees, naive Bayes, and discriminant analysis in the following.

This experiment used three different smartphone models (iPhone Xs Max, Huawei P9, Bluebird) to test the location discernibility of local magnetic fingerprinting. Among them, Bluebird is an industrial custom smartphone for location-based services.

**Figure 14:**
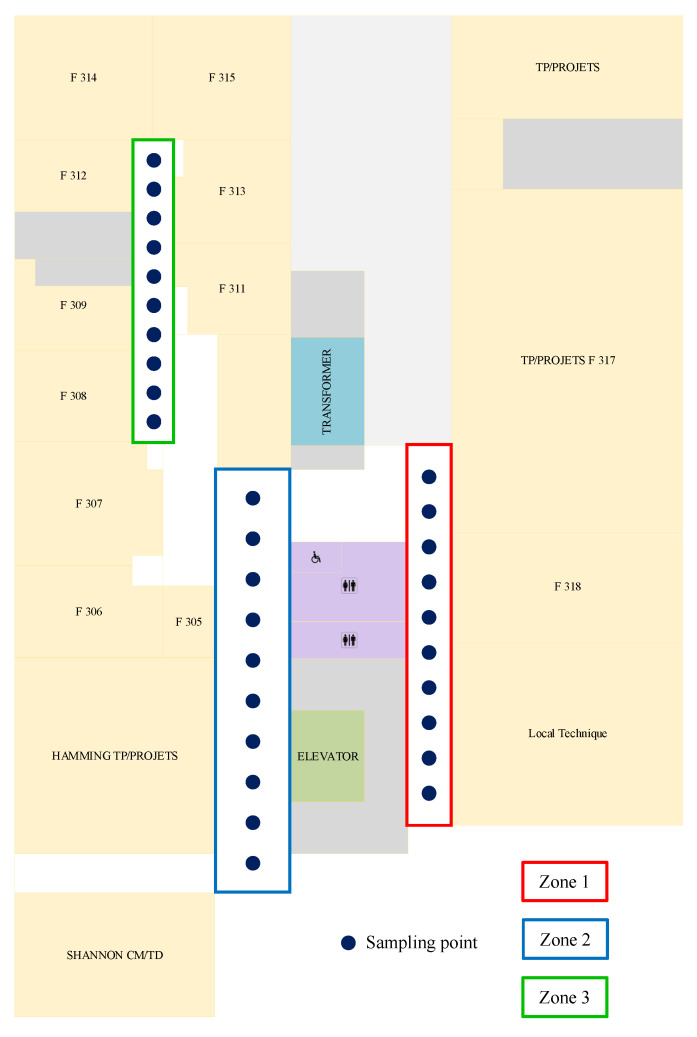
Building of Polytech Orléans—Galilée, Univ. of Orléans with test zone 1, 2 and 3.

The test area is a corridor in the 3rd floor of Polytech Galilée, University of Orléans (shown in Figure 14); zones 1, 2, and 3 each contain 10 sampling points. Firstly, the user stands for 5 s at each point, holding a smartphone to capture a magnetic fingerprint database of 30 points with 100 Hz sampling frequency. Next, the user stands for 1 s at each point to capture test data and calculates the average of these 100 samples; this process was repeated five times to obtain 5 test datasets. Finally, we obtained *n* = 15,000 samples of training data $X_{training}=\left\{x_{1},x_{2}\ldots x_{n}|x_{i}\right\}\in R^{n\times 3}$, and k=150 samples of test data $X_{test}=\left\{x_{1},x_{2}\ldots x_{k}|x_{j}\right\}\in R^{k\times 3}$, respectively. Each sample has a label belonging to class $y=\left\{1,2\ldots 30\right\}$.

In the experiments, the test and training sets were collected in the same direction (along the corridor direction). The sampling interval between two successive points is set to 1.5 m. The height of a smartphone is set at 1.05 m distance from the ground.

### 6.1. Data Pre-Processing

In Section 5, we have already discussed the significance of MF calibration. Hence, in this experiment, the raw MF signal is pre-processed before classification tests. More precisely, the MF measurements are calibrated according to Equation (14), where the rotation matrix $R^{nb}$ comes from the TRIAD algorithm mentioned in [98]. MF measurement on the smartphone’s frame is transformed into the horizontal (denoted as $m_{h}$) and vertical (denoted as $m_{v}$) components [27]. After this transformation, the horizontal and vertical components are “ideally” independent of the user’s direction. Thus, the features of the training and test data can be expressed as:

(15) $$\begin{array}{c} x_{i}=\left\{m_{h},m_{v},magnitude\right\}\in R^{3}, \end{array}$$

The RLOWESS smoothing filter mentioned in Section 4.4 is also used to eliminate anomalies in the smartphone’s MF measurements.

### 6.2. Machine Learning Methods Used for Classification

Magnetic field positioning can be seen as a classification problem. Training data with labels are used to train the classifier in the training phase. Afterward, the new data are fed into the classifier for classification. The machine learning methods used for the tests will be briefly described here.

**Fuzzy*****k*****-nearest neighbor**: The fuzzy *k*-nearest neighbor algorithm [99] is a well-known algorithm for classification. The theory of fuzzy sets was introduced into the *k*-nearest neighbor technique to develop a fuzzy version of this algorithm called fuzzy *k*-nearest neighbor. Not only does the fuzzy algorithm have the advantage of a lower error rate, but the resulting membership also provides a confidence level in the classification. Fuzzy *k*-nearest neighbor was shown to perform well compared to other more complex classification algorithms. The principle of the algorithm is to assign membership as a function of the Euclidean distance vector from the basic *k*-nearest neighbor algorithm and memberships in the possible classes.

Consider *m* feature samples $X=\left\{x_{1},x_{2}\ldots x_{n}|x_{i}\in R^{m}\right\}$ collected from *n* points in the test area (namely N labeled class). The second magnetic test set contains *m* feature samples $y=\left\{y_{1},y_{2}\ldots y_{k}|y_{j}\in R^{m}\right\}$ from *l* points, and we compute the metric

(16) $$\begin{array}{c} \mu_{i}\left(y\right)=\frac{\sum_{j=1}^{K}\mu_{ij}\left(1/{∥y-x_{j}∥}^{2/(m-1)}\right)}{\sum_{j=1}^{K}\left(1/{∥y-x_{j}∥}^{2/(m-1)}\right)}. \end{array}$$

where j=1,2…,K, with *K* being the number of nearest neighbors; i=1,2…,n, with *n* being the number of labeled classes. $\mu_{i}\left(y\right)$ denotes the specified membership of vector *y*, and $\mu_{ij}$ represents the membership of class *i* of the *j*-th vector for the labeled sample set. The fuzzy parameter *m* is used to determine the weights of the distances; here, we set m=2.

**Decision tree**: A decision tree is a non-parametric supervised learning technique consisting of multiple decision rules, all of which are derived from data features. In a decision tree, we call the whole sample the root node, and the process of dividing a node into two or more sub-nodes is called splitting. When a sub-node splits into more sub-nodes, it is called a decision node. Nodes that do not split are called leaves. The process of deleting the sub-nodes of a decision node is called pruning. The decision tree algorithm splits the training set (root node) into subsets, recursively splitting until no pure sub-nodes (leaf nodes) are obtained. The decision tree algorithm requires optimal attributes and thresholds that maximize the splitting criteria (e.g., CART Tree), and the resulting set of splits is optimal. Commonly used decision tree models such as the CART algorithm use Gini’s impurity index, the ID3 algorithm uses Information Gain, and the C4.5 algorithm uses the Gain Ratio [59].

**Naive Bayes**: The naive Bayes classifier is based on Bayes’ theorem. Suppose that *m* feature samples $X=\left\{x_{1},x_{2}\ldots x_{n}|x_{i}\in R^{m}\right\}$ belong to *k* class $y=\left\{y_{1},y_{2}\ldots y_{k}\right\}$. According to Bayes’ rule, $P\left(y_{i}|x\right)$ can be expressed as

(17) $$\begin{array}{c} P\left(y_{i}|x\right)=\frac{P\left(x|y_{i}\right)\cdot P\left(y_{i}\right)}{P(x)}, \end{array}$$

where $P\left(y_{i}\right)$ and $P\left(x\right)$ are known; to estimate the location, we need to find the corresponding location $y_{i}$ that maximizes $P\left(x|y_{i}\right)$. Since the magnetic values obey a Gaussian distribution, i.e., $P\left(x|y_{i}\right)∼N\left(\mu ,\sigma^{2}\right)$, μ and σ are derived from the training set [58].

**Discriminant analysis**: Discriminant analysis methods are well known for learning discriminative feature transformations and can be easily extended to multiple-class cases [100]. Suppose that we have *m* feature samples $X=\left\{x_{1},x_{2}\ldots x_{n}|x_{i}\in R^{m}\right\}$ belonging to *k* class $y=\left\{y_{1},y_{2}\ldots y_{k}\right\}$. The within-class scatter matrix is given as:

(18) $$\begin{array}{c} {\hat{\Sigma }}_{w}=\sum_{i=1}^{n}\sum_{x\in y_{i}}\left(x-\mu_{i}\right){\left(x-\mu_{i}\right)}^{⊤}, \end{array}$$

where $\mu_{i}=\frac{1}{l_{i}}\sum_{x\in y_{i}}x$ and $l_{i}$ is the number of samples in $y_{i}$. The between-class scatter matrix equation is defined as

(19) $$\begin{array}{c} {\hat{\Sigma }}_{b}=\sum_{i=1}^{n}l_{i}\left(\mu_{i}-\bar{\mu }\right){\left(\mu_{i}-\bar{\mu }\right)}^{⊤}, \end{array}$$

where $l_{i}$ is the number of training samples for each class, $\mu_{i}$ is the mean for each class, and $\bar{\mu }$ is the total mean vector given by $\bar{\mu }=\frac{1}{l}\sum_{i=1}^{n}l_{i}\mu_{i}$. The Fisher criterion suggests that the linear transformation w maximizes the ratio of the determinant of the between-class scatter matrix of the projected samples to the within-class scatter matrix of the projected samples [60]

(20) $$\begin{array}{c} J\left(w\right)=\frac{\left|w^{⊤}{\hat{\Sigma }}_{b}w\right|}{\left|w^{⊤}{\hat{\Sigma }}_{w}w\right|}. \end{array}$$

The transformation w can be obtained by solving the generalized eigenvalue problem [100]:

(21) $$\begin{array}{c} {\hat{\Sigma }}_{b}w=\lambda {\hat{\Sigma }}_{w}w \end{array}$$

Discriminant analysis has also its limitations; it assumes a unimodal Gaussian likelihood, and for non-Gaussian distributions, discriminant analysis predictions will not preserve any complex structure of the data. It fails when the discriminant information is not in the mean but in the variance of the data.

**Support vector machine**: Support vector machine classifies data by finding the best hyperplane, which is the hyperplane with the maximum distance between two classes. Consider *m* feature samples $X=\left\{x_{1},x_{2}\ldots x_{n}|x_{i}\in R^{m}\right\}$ belonging to *k* labels $y=\left\{y_{1},y_{2}\ldots y_{k}\right\}$. The *i*-th SVM is trained using all examples with positive labels in the *i*-th class and all other examples with negative labels, and the *i*-th SVM is formulated as follows:

(22) $$\begin{array}{cc} \min_{w^{i},b^{i},\xi^{i}} & \;\frac{1}{2}{\left(w^{i}\right)}^{⊤}w^{i}+C\sum_{j=1}^{l}\xi_{j}^{i}{\left(w^{i}\right)}^{⊤}\\ & {\left(w^{i}\right)}^{⊤}f\left(x_{j}\right)+b^{i}\geq 1-\xi_{j}^{i},\;\;\mathrm{if}\;y_{j}=i\\ & {\left(w^{i}\right)}^{⊤}f\left(x_{j}\right)+b^{i}\leq -1+\xi_{j}^{i},\;\;\mathrm{if}\;y_{j}\neq i\\ & \xi_{j}^{i}\geq 0,\;j=1,\ldots ,l \end{array}$$

where the training data $x_{i}$ are mapped to a higher-dimensional space by a function *f*, $w\in R^{m}$ is a vector representing the direction of the separated hyperplane, b∈R is a constant representing the position of the hyperplane, *C* is the penalty parameter that defines the trade-off between large separation regions and misclassification errors, and $\xi_{j}^{i}$ is a slack variable that allows some samples to be on the wrong side of the separation hyperplane [101,102,103]. According to Equation (22), we can obtain the decision functions

$$\begin{array}{c} {\left(w^{1}\right)}^{⊤}f\left(x\right)+b^{1}\\ \vdots \\ {\left(w^{k}\right)}^{⊤}f\left(x\right)+b^{k}. \end{array}$$

where *x* belongs to the class with the largest value of the decision function.

(23) $$\begin{array}{c} \;\mathrm{class}\;\mathrm{of}\;x\equiv \arg\max_{i=1,\ldots ,k}\left({\left(w^{i}\right)}^{T}f\left(x\right)+b^{i}\right) \end{array}$$

### 6.3. Classification Result

**Figure 15:**
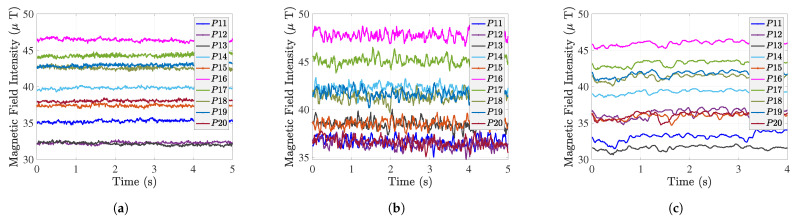
Smartphone training set in zone 2. (**a**) iPhone Xs Max. (**b**) Huawei P9. (**c**) Bluebird.

The test zones are shown as three rectangles in Figure 14, with 10 test points in zones 1, 2, and 3, respectively. The green area is the elevator, which is a source of magnetic interference. Figure 15 shows the “calibrated” MF intensity at 10 points in zone two as an illustrative example to visually analyze the MF magnitude fingerprints in this zone. The Huawei device has a more significant variance in the MF intensity than the other two smartphones, with more overlap between the signals. Bluebird’s database shows that there is an overlap between P12, P15 and P20, P18, and P19. The iPhone has a slight overlap between P12 and P13.

As mentioned above, the training dataset sampling time for this experiment is 5 s, so there are 500 training samples. More training data can better train the prediction model. The test data are the average of the test dataset samples (100 samples in total).

The accuracy is calculated according to Equation (24),

(24) $$\begin{array}{c} \;\mathrm{Accuracy}\;=\frac{Number\;of\;all\;correctly\;labeled\;points}{Number\;of\;all\;points}. \end{array}$$

**Figure 16:**
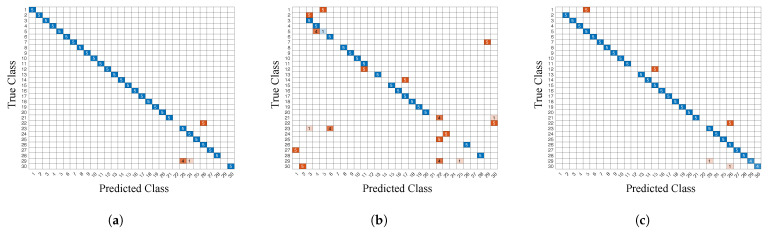
Confusion matrix for KNN methods with different smartphones. (**a**) iPhone Xs Max. (**b**) Huawei P9. (**c**) Bluebird.

First, we test the training and test sets from the same smartphone separately, and the results are shown in Table 5. The positioning accuracies of the iPhone Xs Max and Bluebird are relatively similar (close to 80 or above), basically meeting the needs of essential indoor positioning. In contrast, the positioning accuracy of the Huawei P9 is significantly lower than the other two smartphones. Figure 16 shows the confusion matrix for the three smartphones using the KNN method, blue indicates correctly classified points, other colors indicate incorrectly classified points.Huawei P9 performs better in zone 2 (landmark 11 to landmark 20) and does not have high positioning accuracy in zones 1 and 3. The elevator in the green area of Figure 14 is a source of magnetic field interference, making this area even more special. The results from Huawei P9 show that the interference source improves the positioning performance (i.e., it enhances the differences between the MF features). The variances of the MF measurements for the iPhone Xs Max, Bluebird, and Huawei P9 are 0.04 μT, 0.12 μT, and 0.21 μT, respectively. As the intensity of the magnetic field varies smoothly between 23 μTesla and 62 μTesla [87], the larger the variance, the more likely it is to overlap with other signals within a limited variation interval, which degrades the positioning performance. Due to the significant variance of the MF measurement, Huawei’s positioning accuracy is lower than the other two smartphones.

Next, we use the iPhone Xs Max for the training set to obtain the prediction model and test the other two smartphones separately. Table 6 illustrates the positioning accuracy. We can see that the positioning accuracy decreases when the magnetic fingerprinting database and the test data are inconsistent compared to Table 5. We already saw earlier that the Bluebird’s MF measurement is significantly different from the other two smartphones. Nonetheless, using the calibrated Bluebird’s MF measurement, we can also achieve accuracy of approximately 50%, which means that we have the opportunity to use the iPhone to create a magnetic field fingerprint database that other smartphones can use for positioning. We can also see that KNN, discriminant analysis, and SVM have better positioning accuracy than decision tree and naive Bayes in this experiment.

## 7. Conclusions

With the widespread use of smartphones, ubiquitous magnetic fields are attracting researchers’ interest. The use of smartphones to measure magnetic fields for indoor positioning has significant advantages: infrastructure-free, temporal stability, and spatial uniqueness.

However, there are also significant challenges highlighted by our study:

- Firstly, the use of MF data requires the processing of device heterogeneity. The magnetometers with different specifications used by smartphone manufacturers result in different MF measurements. Hence, MF fingerprinting would require the use of smartphones/magnetometers which have similar characteristics to ensure the efficiency of such a positioning approach.
- Data pre-processing is necessary in order to exploit the MF data. This includes filtering out the outliers that affect the magnetometer measurements (in this work, we propose the RLOWESS algorithm to smooth the MF measurements). It also includes the calibration of the magnetometer, which is necessary to eliminate soft and hard iron influences.
- The magnetic signatures of heterogeneous smartphones on the same path have the same pattern but do not overlap. As the X and Y axes of the magnetic field are direction-dependent, the MF intensity of the smartphone fluctuates as it rotates around the Z axis, which is challenging for magnetic field map construction.
- Calibration tests were carried out with different smartphones in specific locations at given dates. It was found that the calibration parameters of the smartphones depend only on its specifications and not on the environment. There is no need to re-estimate the calibration transform periodically or for different locations.
- The MF collected by one smartphone is calibrated as a fingerprint database, and other smartphones can use this MF fingerprint database for positioning. This method can somewhat solve the MF positioning problem of heterogeneous devices. However, we can still see that the positioning accuracy of heterogeneous devices is significantly lower than that of homogeneous devices.
- Interference sources may enhance the specificity of local MF fingerprints (e.g., proximity to fridges, lifts, metal doors). In the above experiment, the Huawei P9’s positioning accuracy was significantly higher in zone 2 than in the other two zones.
- Despite these challenges, MF data can be used as a complementary method to improve the positioning accuracy of hybrid positioning solutions (e.g., in combination with Wi-Fi, Bluetooth, etc.).

## Figures and Tables

**Table 1:** Advantages and disadvantages of indoor positioning technologies.

Positioning Technology	Coverage Range	Positioning Accuracy	Advantages	Disadvantages
Ultrasound [17]	∼10 m	Meters	Slow propagation speed (around 340 m/s); Limited influence of the surroundings and good penetration of walls.	Multipath effects and attenuation.
Wi-Fi [18]	∼35 m	5 m∼15 m	Widely distributed hot spots; Low access conditions; High flexibility.	High fingerprint collection effort; Vulnerable to access point changes; Fluctuation of Wi-Fi signal; Radio mismatch problems; Heterogeneity of Wi-Fi devices; Noise and multipath distortion.
Bluetooth [19]	∼10 m	1∼5 m	Low reception range; Low energy consumption.	Low positioning accuracy; Prone to noise.
UWB [20]	Few meters	10∼30 cm	Immune to interference; High accuracy; High multipath resolution; Large bandwidth.	Shorter range; Extra infrastructure requirement; High cost for users.
Visible light [21]	Line of sight condition	10 cm∼2m	Device-free; Security; Less infrastructure changes in passive devices; Energy efficiency.	High infrastructure changes on the transmitter side; The burden on the user; Complex infrastructure.
Vision (camera) [22]	Line of sight condition	0.01∼1 m	High positioning accuracy; unaffected by the external environment; Strong anti-interference capability.	Complex algorithms; High power consumption; Sensitive to light conditions; Expensive and lacks wide applications.
Inertial navigation [23]	Hundreds of meters	2 m	Low cost; Easy to deploy.	Subject to the accuracy of inertial sensors; Accumulation of drift and deviation errors.
Magnetic field [24]	∼	1∼5 m	Infrastructure-free; Temporal stability; Uniqueness due to ferromagnetic disturbance; Tolerance to moving objects.	Low discernibility; Need for frame transformation; Heterogeneous device.

**Table 2:** Magnetometer information and operating systems for heterogeneous smartphones.

Smartphone	System Version	Magnetometer Model	Sensor Vendor	Description
Huawei P9	Android 8.0	AK09911	AKM	3-axis, 14-bit; 0.6 μT/LSB; Range: 4900 μT
Redmi Note 10 Pro	Android 11	AK0991x	AKM	3-axis, 14-bit; 0.15 μT/LSB Range: 4900 μT
Samsung S9	Android 9.0	AK09916C	AKM	3-axis, 16-bit; 0.15 μT /LSB; Range: 4670 μT
Bluebird	Android 6.0	Mmc3416x	MEMSIC	3-axis, 16-bit; 0.05 μT /LSB; Range: 1600 μT
iPhone Xs Max	iOS 15.3.1	∼	∼	∼

**Table 3:** Summary of MF intensity statistic characteristics.

Time	Device	Mean	Std	Kurtosis	Skewness
22 January 2021	iPhone Xs Max	46.87	0.25	2.72	−0.06
	Huawei P9	50.00	0.52	3.87	0.32
	Bluebird	122.24	1.94	506.21	−0.04
4 February 2021	iPhone Xs Max	47.63	0.37	3.09	−0.62
	Huawei P9	49.01	0.53	3.97	0.37
	Bluebird	125.19	1.79	1764.79	12.33

**Table 4:** MF intensity variance comparison.

	D1	D2	D3	D4	D5	D6
Original MF Variance	3.67	0.24	0.74	0.20	8.03	0.20
Filtered MF Variance	0.01	0.19	0.01	0.16	0.07	0.11

**Table 5:** Accuracy comparison of homogeneous devices with different positioning methods.

Smartphone	KNN	Decision Tree	Naive Bayes	Discriminant Analysis	SVM
iPhone	93.3%	76.7%	76.7%	88.0%	86.0%
Huawei	53.3%	40.7%	40.7%	42.7%	52.0%
Bluebird	88.7%	82.0%	82.0%	89.3%	88.0%

**Table 6:** Accuracy comparison of heterogeneous devices with different positioning methods. (iPhone Xs Max is the training set; Huawei P9 and Bluebird are the test sets, respectively).

Smartphone	KNN	Decision Tree	Naive Bayes	Discriminant Analysis	SVM
Huawei	59.3%	53.3%	53.3%	47.3%	46.0%
Bluebird	59.3%	44.7%	44.7%	55.3%	55.7%

## Data Availability

Not applicable.
